# Effects of high-amylose maize starch on the glycemic index of Chinese steamed buns (CSB)

**DOI:** 10.1016/j.heliyon.2022.e09375

**Published:** 2022-05-04

**Authors:** Noraidah Haini, Lee Jau-Shya, Ramlah George Mohd Rosli, Hasmadi Mamat

**Affiliations:** Faculty of Food Science and Nutrition, Universiti Malaysia Sabah, Jalan UMS, 88400, Kota Kinabalu, Sabah, Malaysia

**Keywords:** Resistant starch, Chinese steamed bun, Postprandial glycemic response, Glycemic index

## Abstract

The incorporation of resistant starch (RS) in food has gained importance to be a good replacement for digestible carbohydrate. This study examined the effect of compositing RS (high-amylose maize starch (HM)) as wheat flour substitute (30%) in Chinese steamed bun (CSB) formulation on postprandial glycemic response in healthy human subject. In this single-blind and cross-over experimental trial, a total of 15 female participants (mean age = 31.5 ± 3.9) were randomly assigned to receive CSB containing 30% HM (HM30) or control CSB (without HM) with their blood glucose were recorded throughout the test. The blood glucose concentrations recorded for HM30 were significantly lower than control CSB at 15 min (6.03 vs. 7.04 mmol/L, *p* = 0.041), 30 min (6.93 vs. 7.76 mmol/L, *p* = 0.021), 45 min (6.21 vs. 7.55 mmol/L, *p* = 0.032), 60 min (5.68 vs. 6.26 mmol/L, *p* = 0.038), and 90 min (5.08 vs. 5.73 mmol/L, *p* = 0.022). The 2-h postprandial glucose was significantly lower in HM30 (iAUC = 105.2 mmol x min/L) than the control (186.1 mmol x min/L). The low GI property of HM30 (GI = 39.11 ± 5.6) did not cause sudden rapid increase in blood glucose concentration as observed in medium-GI control CSB (GI = 69.18 ± 9.8). This study suggests that adding 30g of HM decreased the glycemic index of CSB in healthy female adult.

## 1 Introduction

Glycemic index (GI) was first introduced in 1981 as a classification for blood glucose-raising ability of digestible carbohydrates. GI is defined as the incremental area under the blood glucose curve (iAUC) following the intake of a 50 g carbohydrate portion of a test food and expressed as percentage of response compared with 50 g digestible carbohydrate of a reference food taken by the same subject (Jenkins et al., 1981). Food carbohydrates that are slowly digested belong to low-GI food (GI < 55), whereas those with intermediate rate of digestion pattern belong to medium-GI food (GI < 70) (Zhu, 2019). Food carbohydrates that are rapidly digested are found in high-GI food (GI > 70). High-GI diets have been associated with a range of metabolic diseases and health condition, typically diabetes and obesity (Zhu and Li, 2019).

Previous studies reported the ability of low-GI food to improve blood glucose control for patients with diabetes (Ojo et al., 2018), aid in weight management (Zafar, 2018), and reduce the risks of cardiovascular diseases (Clar et al., 2017). The consumption of low-GI foods can increase satiety, reduce appetite, delay hunger, and reduce excessive energy intake in adults (Stewart et al., 2018). Given the limitations of high cost and potentially hazardous side effects of treatment drugs, the potential of using functional food for preventive measures is currently investigated (Maziarz et al., 2017). Lowering the GI of food could therefore be an alternative in aiding and managing general health programs.

With the wide spread of awareness of the GI concept, efforts have been carried out to reduce the GI of normally high-GI food (Wang et al., 2018), such as bakery and pastry products (Zhu and Li, 2019). Chinese steamed bun (CSB) is a type of high-GI food. Given its high GI value, CSB is sometimes used in glycemic test as a reference food apart from the normal glucose solution (Zhu, 2019). In Malaysia, CSB is a staple food, and an average of 17.11% of Malaysian population eat one serving of CSB every day (Kasim et al., 2018). As CSB is highly consumed and a relevant source of available carbohydrates, scholars have focused on reducing the GI value of CSB by using a functional ingredient known as type-2 resistant starch (RS) such as high-amylose maize starch (HM) (Abdul Shukri et al., 2017; Haini et al., 2021).

Substantial evidence indicates that HM is associated with blood glucose-lowering effects in animal models (Shen et al., 2011) and human clinical trials (Marlatt et al., 2018; Stewart et al., 2018). Nonetheless, limited studies are available on the use of HM-enriched CSB for glycemic response effect. According to Zhang et al. (2006), incorporating HM in bread delayed gastric emptying and thus reduced the GI (low GI: 47) and delayed the glycemic response in patients with diabetes. To date, no research has been published on the GI of HM-enriched CSB of human subjects. Henceforth, this study aims to determine the GI of HM-enriched CSB. The potential use of HM in CSB is explored to serve as a novel CSB product with potentially low GI value.

## 2 Materials and methods

### 2.1 CSB formulation

CSB formulations were modified from the study of Shukri et al. (2017). All the ingredients used are in flour weight basis. In brief, 100 g of steamed bun flour (10.5% protein, 75% carbohydrate and 0.8% fat on dry basis) (Sabah Flour Mill, Malaysia), 30% HM (Hi-Maize® 260) (Ingredion, Australia), 8% castor sugar (Kings, Malaysia), 2% dry yeast (Saf-Instant®, France), 1% salt (Servay, Malaysia), and 0.5% baking powder (Royal®, USA) were mixed at speed 1 (60 rpm) for 5 min (Kitchen Aid, Taiwan) and added with 66.76 % water. The dough was added with vegetable shortening (1.5%) (Crisco®, USA), mixed to uniformity (speed 5, 165 rpm for 10 min and speed 7, 265 rpm for 10 min), and cut into several pieces (50 g per piece). Each piece of dough was rounded, molded manually, and then proofed (GX-26A) for 45 min at 38 °C and 85% relative humidity. The proofed dough was steamed for 15 min by using a steam tray and boiling water then cooled to room temperature for 2 h.

### 2.2 GI experimental test

#### 2.2.1 Ethical issues

The study was ethically approved by the Medical Research Ethics Committee of Universiti Malaysia Sabah [UMS/FPSK6.9/100-6/1/95; JKEtika 3/18(7)]. The informed consent form was signed by the subjects to ensure their voluntary enrolment to participate in the study. Transportation was provided for subjects who encountered difficulty in arriving and departing from the Faculty of Food Science and Nutrition, Universiti Malaysia Sabah. Breakfast was provided for each subject upon completion of each test as gratitude for their voluntary participation.

#### 2.2.2 Study location

The study was conducted in Nutrition and Dietetic Clinic at Faculty of Food Science and Nutrition, Universiti Malaysia Sabah. All tests were carried out on selected weekdays at 8.00 a.m.–11.00 a.m. from 1 March to 30 August 2020. Under supervision of the investigator, the subjects consumed reference and test food on their own (without additional flavoring) and served with plain water.

#### 2.2.3 Study design

This study adopted a cross-over design, and blood glucose concentration was determined at specific time points. The subjects were required to attend seven sessions throughout the study, with three sessions consisting of reference foods and the two other sessions for each test foods, namely as HM-enriched CSB (HM30) and control CSB.

#### 2.2.4 Reference food and test food serving size

The reference food was Glukusking© Dextrose Monohydrate (Hurix, France). The available carbohydrate content of Glukusking© Dextrose Monohydrate was obtained through the nutritional information provided from the food manufacturer. As 100 g of Glukusking© Dextrose Monohydrate contains 91 g of dextrose monohydrate, the glucose solution was prepared by dissolving 54.9 g of Glukusking© Dextrose Monohydrate in 250 mL of water.

A test portion containing 50 g of available carbohydrate was required to determine GI of food (Brouns et al., 2005). In the GI test, the available carbohydrate was measured as (Total starch x 1.1) – (RS x 1.1) + (total disaccharides x 1.05) + total monosaccharides – dietary fibre (Brouns et al., 2005). Both the test food, namely, control CSB and HM30, were cooked in the Bakery Technology Laboratory of Faculty of Food Science and Nutrition, Universiti Malaysia Sabah. Control CSB and HM30 were served to the subjects in a 50 g available carbohydrate portion. A standard amount of 250 mL of water was served together with the test food. The fluid volume was standardized as the amount of water ingested that influenced the pattern of glycemic response (Brouns et al., 2005). The nutrient composition and serving amount of the test food are shown in Table 1.

**Table 1:** Nutritional composition for each serving amount of control CSB and HM30.

Test foods	Weight (g)	Energy (kcal)	CHO∗ (%)	Protein (%)	Fat (%)	Total dietary fibre (%)	Resistant starch (%)
HM30	100	268	46.78	6.16	1.02	1.27	22.08
Serving amount	106.88	286.43	50.0	6.58	1.09	1.36	23.60
Control CSB	100	290.80	96.43	9.41	1.21	0.63	0.38
Serving amount	51.85	150.78	50.0	4.88	0.63	0.33	0.20

∗ CHO refers to available carbohydrate (Brouns et al., 2005).

#### 2.2.5 Subject recruitment

All female subjects (n = 15) were recruited from Universiti Malaysia Sabah through advertisement in social network and word by mouth. Only female subjects were chosen considering that glycemic response differed between males and females (Ministry of Malaysia, 2015). In reviews on gender differences in the regulation of glucose homeostasis, Tramunt et al. (2020) and Greenhill (2018) had reported that females are more insulin sensitive than men. Furthermore, Mauvais-Jarvis (2018) also reported that glucose insulin sensitivity index was higher in women than in men, even after adjustment for age and BMI. Meanwhile, Ishii et al. (2016) found that the GI of glucose and sucrose in women were significantly higher than that was found for men. Those findings indicated that the resulting postprandial glucose are different between males and females, therefore, the authors want to exclude confounding factors such as hormonal and insulin sensitivity due to gender differences which could affect the resulting postprandial glycemic response in present study.

All potential subjects were screened to ensure that they fulfilled the following criteria:

1. Not on medication, no history of metabolic disorders, and had no known chronic diseases, such as diabetes, malabsorptive disorder, untreated hypertension, cardiovascular diseases, kidney diseases, liver diseases, or gastrointestinal disorders
2. Had BMI between 18.5–23.0 kg/m^2^
3. Had fasting blood glucose concentration <6.1 mmol/L
4. Non-smoker
5. Not pregnant or lactating
6. Consumed <7 units/week of alcohol beverages if any

Source: Brouns et al. (2005); Vega-López et al. (2007).

### 2.3 Study protocol

The protocol for this study was in line with procedures recommended by the FAO/WHO (1998), Brouns et al. (2005), and Malaysian Guidelines for Good Clinical Practice (Ministry of Health Malaysia, 2011). All the subjects went through the study protocol on seven separate occasions (two for each test food and three on the reference food) with a three-day wash out period (Brouns et al., 2005) in between each test to minimize the carry-over effects. The day prior to each test, the subjects fasted for 10–12 h and refrained from any unusual and intense physical activities (Wolever, 2004). One-day dietary record was obtained by the subjects prior to the first test to maintain almost the same meal pattern prior to each glycemic response test based on the dietary record.

On the day of the actual test, the subjects arrived at Nutrition and Dietetic Clinic, Faculty of Food Science and Nutrition, University Malaysia Sabah by using the least strenuous transport possible. All tests were carried out in the morning in fasting state, which was the most stable state concerning the non-existence of any possible intra-individual differences over influences of previous meal (Wolever, 2004). Blood concentration was determined in fasting state (0 min) to serve as the baseline for glycemic response curve.

All the test food and reference food were prepared beforehand. HM30 and control CSB were prepared 4 h earlier in the morning on the same test day itself (as a way to minimize the effect from starch retrogradation after cooking). For glucose drink, it was prepared 2 h before serving. The order of test food was randomized, and all the allocation sequence was unpredictable. All the subjects were required to finish the test or reference food within 15 min in each session. The time allocated was set to allow for convenience during consumption and to minimize gastric emptying rate effects and discomfort among the subjects. Subsequent blood sampling was carried out at 15, 30, 45, 60, 90, and 120 min after the first sip of reference food or first bite of test food. Throughout the 2 h period, the subjects could carry out light activities such as reading, watching movie, or typing. However, they were not allowed to leave the laboratory without permission from the investigator.

### 2.4 Chemical compositions of HM30 and control CSB

Proximate composition analyses were conducted according to AOAC (2013) for fat, protein, and carbohydrate. Total dietary fibre (TDF) was measured using a Megazyme TDF kit (AACC, 2000). The calorific content (kcal/100 g) was calculated by multiplying crude protein, crude fat, dietary fibre, and available carbohydrate contents by factors of 4, 9, 2 and 4, respectively. Total energy was expressed in terms of kilocalories (Kcal) unit (Ministry of Health Malaysia, 2011). Total starch and resistant starch were determined using the Megazyme Kit (Megazyme, Ireland) based on AACC (2000), Method 32–40.01 and 76-13.01, respectively.

### 2.5 Anthropometry measurement

Anthropometry measurement for height and weight was conducted to calculate body mass index (BMI) (Ministry of Health Malaysia, 2015). A stadiometer (SECA 213 portable measuring rod; United Kingdom) was used for height measurement, whereas an electronic weighing balance was used for weight measurement. BMI was obtained by dividing weight in kilogram by height in meters squared (Ministry of Health Malaysia, 2015).

### 2.6 Blood glucose measurement

Blood glucose concentration was measured using glucometer (Accu-Chek® Active Blood Glucose Monitor, USA) and recorded in mmol/L unit. Capillary blood was used instead of venous blood because the within subject variations using capillary sample were lower than those using venous sample (Wolever, 2004).

### 2.7 Blood pressure measurement

Systolic (SBP/mmHg), diastolic blood pressure (DBP/mmHg), and pulse (beat/minute) were measured by an automated sphygmomanometer (OMRON SEM-1 Automatic Blood Pressure Monitor 20100609672LF, Japan). The subjects were reminded to wear comfortable and loose clothing because the measurement was taken on the upper side of their arms. At each visit, the subjects in their fasting state were asked to be seated, remained silent, relaxed, and rested at least 5 min before measurement, with both feet flat on the floor and their back and arm supported. Prior to measurement, blood pressure readings were taken in both arms. The arm that gave higher systolic record was used for all future measurements.

### 2.8 Calculation of glycemic index

Several methods have been proposed for the calculation of GI of food (Brouns et al., 2005). The method used in the study was recommended by the FAO/WHO (1998). The method requires calculation of incremental area under the curve (iAUC) with area beneath the fasting blood glucose concentration being ignored. iAUC was determined geometrically using the trapezoid rule.

The GI for the test food was calculated as the mean of individual ratios. The individual GI obtained by each subject was averaged over all the subjects to obtain the actual GI of the test food.

Glycemic index (GI) of food was calculated using the formula shown below:

(1) $$GI=\frac{iAUC\;of\;test\;food\;containing\;50\;g\;available\;carbohydrate}{Mean\;iAUC\;of\;test\;food\;containing\;50\;g\;available\;carbohydrate}\times 100$$

Source: Brouns et al. (2005); Wolever (2004); Jenkins et al. (1981).

### 2.9 One-day dietary record

Prior to the start of the first session of the GI test, the subjects were taught the correct way of recording the one-day dietary record and were required to document all the food and drinks they consumed one day before the first test. The amount of food and drinks consumed was recorded using household measurement tools (example: teaspoons, tablespoons, cups). The brand and name of foods, preparation methods, and recipes of combination food were included to enhance the details of food. During the first session of the test, each subject's food record was reviewed by the investigator to clarify the entries and probe for forgotten food. The dietary record was given back to the subjects at the end of first test session. The subjects were encouraged to consume the same type of meals as recorded in the dietary record the day before any subsequent tests to minimize the influence of the interaction of different meals on the accuracy of the test results.

### 2.10 Determination of postprandial glycemic response and glycemic index (GI)

Data analysis for the GI study was carried out using SPSS software (Version 25, IBM Corp., USA). The results for determination of postprandial blood glucose concentration and glycemic index on test food were presented as mean ± standard deviation (SD). Before statistical analysis, the normality of the data was tested using Shapiro–Wilk statistics. Statistical significance was set at *p* < 0.05.

The glycemic responses for reference food (glucose) and test food (HM30 and control CSB) were displayed with y-axis labelled as blood glucose concentration (mmol/L) and x-axis labelled as time (min) for calculation of iAUC (FAO/WHO, 1998). The mean iAUC of the test and reference food was compared using ANOVA and Tukey's B post-hoc for multiple comparisons. Paired sample t-test was used to determine significant differences in postprandial glycemic responses between the two test foods.

The GI values of HM30 and control CSB were calculated using Eq. (1). Paired sample t-test was used to determine whether the GI of the two test foods were significantly different. The levels of within- and between-subject variations of the three reference tests were assessed by determining the percentage of the coefficient of variation (CV).

## 3 Results and discussion

### 3.1 Subject characteristics

Healthy subjects (female = 15; aged 25–38 years) who had mean BMI of 21.9 ± 2.3 kg/m^2^ were recruited and interviewed. The BMI of all subjects was within the normal BMI range between 18.5 – 23.0 kg/m^2^, and their participation in the study was approved (Ministry of Health Malaysia, 2015). Brouns et al. (2005) reported that a study on 15 subjects will provide reasonable degree of blood glucose lowering effect and precision. The characteristics of the subjects are shown in Table 2.

**Table 2:** Subject characteristic (n = 15).

Parameter∗	Mean ± SD
Age	31.5 ± 3.9
Female	15
BMI (kg/m2)	21.92 ± 2.4
SBP (mmHg)	114.8 ± 1.7
DBP (mmHg)	79.1 ± 1.1
Pulse (beats/min)	72.0 ± 1.2
Glu (mmol/litre)	4.64 ± 0.62
TC (mmol/litre)	4.78 ± 0.71
Energy intake	1425.2 ± 89.9

∗ BMI – Body Mass Index, SBP – Systolic Blood Pressure, DBP – Diastolic Blood Pressure, Glu – Glucose, TC – Total Cholesterol.

### 3.2 Glycemic response for reference and test food

Table 3 shows the blood glucose concentration of subjects upon consumption of reference and test food. No significant differences (*p* = 0.277, *p* < 0.05) were observed in fasting blood glucose (FBG) concentration for reference and test food. FBG concentration of reference food was included as a covariate to control the between-subject differences at the baseline. Table 3 shows the mean blood glucose concentration of the subjects at 0, 15, 30, 45, 60, 90, and 120 min upon consumption of reference food (glucose) and test food. The blood glucose concentration significantly increased within 15 min after consumption of reference and test food (*p* = 0.001, *p* < 0.05). The blood glucose concentrations peaked at 30 min upon consumption of reference and test food, with reference food achieving the maximum glycemic response. Between the test food, the peak value of HM30 was significantly lower (*p* = 0.039, *p* < 0.05) than that of control CSB. The blood glucose concentrations recorded for HM30 were significantly lower at 15 min (*p* = 0.041, *p* < 0.05), 30 min (*p* = 0.021, *p* < 0.05), 45 min (*p* = 0.032, *p* < 0.05), 60 min (*p* = 0.038, *p* < 0.05), and 90 min (*p* = 0.022, *p* < 0.05) compared with those for control CSB.

**Table 3:** Blood glucose concentration (mmol/L) of subjects (n = 15) at fixed time point upon consumption of reference food and test food.

Time (min)	Reference food (glucose)	HM30	Control CSB
0	4.85 ± 0.10	4.99 ± 0.17	5.02 ± 0.17
15	7.48 ± 0.13	6.03 ± 0.16	7.04 ± 0.23
30∗	8.84 ± 0.24	6.93 ± 0.15	7.76 ± 0.24
45	8.50 ± 0.22	6.21 ± 0.26	7.55 ± 0.28
60	7.60 ± 0.19	5.68 ± 0.17	6.26 ± 0.23
90	6.57 ± 0.18	5.08 ± 0.22	5.73 ± 0.25
120	5.56 ± 0.20	5.07 ± 0.23	5.58 ± 0.21

Data expressed as mean ± SD.

∗ The peak in blood glucose concentration upon consumption of reference food and test foods.

In healthy adults, blood glucose concentration started to rise within 15 min after consumption of food and peaked at around 30–45 min, depending on the timing, quantity, and composition of food (Brand-Miller et al., 2009). Massive hyperglycemia after consumption of glucose solution was responded by immediate and maximal outpouring of insulin (Gower et al., 2016). Consumption of control CSB provided faster postprandial blood glucose concentration peak compared with HM30. The rise in blood glucose could be due to steaming, where starch in control CSB is fully gelatinized, making the starch more readily digestible (Huang et al., 2011) and resulting in faster glycemic responses (Zhu and Sun, 2019). It was also worth noting that HM30 which had higher RS content compared to control CSB produced a delayed peak at 30 min. The presence of higher amount of resistant starch limiting the diffusion and absorption of glucose into intestinal cells (Fardet et al., 2006).

In normal occasion, within 2 or 3 h, the blood glucose concentration returns to the baseline level and sometimes decreases below the baseline level (Brand-Miller et al., 2009). As shown in Table 3, the blood glucose concentration of all subjects decreased within 2 h. After consumption of HM30, the blood glucose concentration decreased and returned almost to the baseline level compared with control CSB and reference food within 120 min. Interestingly, control CSB did not produce mean capillary blood glucose concentration below the baseline fasting levels, which is often shown in studies utilizing white bread (Foster-Powell et al., 2002).

### 3.3 Incremental area under the curve (iAUC) for reference and test food

The glycemic responses of the subjects toward reference and test food were calculated using iAUC starting from the baseline, with area below the baseline excluded. Table 4 shows the iAUC for reference food (glucose) and test food. The iAUC of glucose (269 mmol x min/L) was slightly higher than those in previous studies on GI tests among Malaysian subjects (224.8–259 mmol x min/L) (Robert et al., 2008). However, in a study that investigated the glycemic response of white bread (Burton and Lightowler, 2006), the iAUC of glucose (279 mmol x min/L) was comparatively nearer to the that in the present study. Meanwhile, the iAUC values of HM30 (105.2 mmol x min/L) and control CSB (186.1 mmol x min/L) were significantly lower (*p* = 0.001, *p* < 0.05) than that of the glucose and were lower than that reported for white CSB (Lau et al., 2015; Liu et al., 2017). According to Zhu (2019), the resulting iAUC of white CSB depends considerably on the ingredient used and CSB volume. The iAUC values of the test food were significantly different (p = 0.001, *p* < 0.05) from the iAUC of glucose. The comparison of iAUC between HM30 and control CSB showed the significantly lower intake of HM30 (*p* = 0.021, *p* < 0.05).

**Table 4:** IAUC^1^ of reference food and test food (n = 15).

Time (min)	Glucose	HM30	Control CSB
Mean ± SD	269.0 ± 42.9	105.2 ± 31.1	186.1 ± 55.7
SEM	12.8	14.2	21.7
Minimum	216.1	48.0	151.6
Maximum	377.0	149.6	218.3
Lower CI^2^	245.8	78.7	152.5
Upper CI^2^	312.3	131.9	210.3
CV (%)	16	29.6	30

1 iAUC unit value of mmol x min/L.

2 CI denotes 95% confidence interval for mean.

According to Wolever et al. (2008), BMI and ethnicity are associated with glycemic responses. For example, the glycemic responses following glucose ingestion were greater in Europeans than in Asians (Kataoka et al., 2013). The slightly higher reference iAUC obtained in the present study might be due to differences in the genetics and physiology of individuals who participated in the GI trial (Zhang et al., 2006). Nevertheless, Brouns et al. (2005) reported that even if glycemic responses differ, the GI value, when calculated, would be similar or average out to a certain range of values because GI value is eventually the property of the food.

### 3.4 Within- and between-subject variations in glycemic responses for reference food

Glycemic response can vary substantially between the same individual even when the same investigator participates in the study and the preparation of reference food is uniform (Hirsch et al., 2013). In the present study, the glycemic response for reference food exhibited within- and between-subject variations. Within-subject variation was assessed by having the subjects repeat the same reference food (glucose solution) on different days.

The glycemic responses yielded by the same reference glucose varied from day-to-day within subjects, with a mean of 21.73% ± 14.16%. The between-subject CV for reference glucose (16%) was lower than that for HM30 (29%) and control CSB (30.0%), which could be due to the three times replication of the glucose test. Most publication suggested that the reference food should be tested three times for each subject to minimize day-to-day variation (Hirsch et al., 2013; Bodinham et al., 2014).

The data for within- and between-subject variations were compared with previous works on healthy subjects. The within-subject variation of reference CV should be less than 30% to obtain accurate GI result for a particular food (Wolever et al., 2008). According to Kaur et al. (2020), the within-subject variation in the glycemic responses of healthy subjects consuming 50 g of glucose was 25.0 ± 12.0%, while the between-subject variation was 26.4%. This finding is almost consistent with the within-subject variations reported for the present study (21.73), and the between-subject variation was comparatively lower by approximately 39% (16%).

For test food, the between-subject CV for the glycemic response of white CSB ranged from 25.2% to 56% (Lau et al., 2015; Liu et al., 2017). In the present study, the between-subject CV of control CSB was 30.0% and was within the range. According to Zhang et al. (2006), a reduction in 20%–25% of CV was obtained when control white CSB was tested more than once. However, due to low within-subject CV for glucose, a good estimate of the glycemic response to test food was obtained through double test for each test food.

Although the unpredictability of individual responses poses limitation to the study, the reliability of the glycemic responses for reference food was acceptable because the values of within-subject variability were relatively closer to those reported by Wolever et al. (1985). The values for between-subject variability of HM30 and control CSB were within the range reported previously (Yang and Zhao, 2012). Most variation of GI value in food is due to within-subject variation, and between-subject variation has no significant effect on acquiring the GI value of food (Wolever et al., 2003). The within-subject and between-subject variations of reference food indicate that glycemic responses are not only affected by the composition of foods but also by uncontrollable variables in healthy individuals, such as absorption rate, stress-induced changes in gastrointestinal motility, and decrease in insulin sensitivity due to sleep deprivation (Marlatt et al., 2018).

### 3.5 Glycemic index of test food

The measured GI values of HM30 and control CSB are shown in Table 5. HM30 (GI = 39.11) belonged to low-GI category, whereas control CSB (69.18) belonged to medium-GI food, borderline to high-GI category. No outliers (individual GI values greater than or lesser than 2SD of the mean GI values) were found. Therefore, all the results from 15 healthy subjects were included in data analysis. The mean GI for HM30 was significantly lower (*p* = 0.021, *p* < 0.05), with 43.5% lower GI units than control CSB. The CV values for GI of HM30 and control CSB were 26.1% and 27.8%, respectively, consistent with the calculated CV range of 12%–75.2% in published studies using healthy subjects (Vega-López et al., 2007).

**Table 5:** Measured GI values of test foods.

Time (min)	HM30	Control CSB
Mean ± SD	39.11 ± 10.2	69.18 ± 21.3
GI classification	Low GI	Medium GI
SEM	5.6	9.8
Minimum	18	40
Maximum	53	88
Lower CI^1^	28.1	36.0
Upper CI^1^	47.4	73.8
CV (%)	26.1	27.8

1 CI denotes 95% confidence interval for mean.

In previous studies, the GI value is highly correlated with the actual incremental blood glucose concentration at 30, 60, and maximum amplitude of glucose excursion at 90 min (Brand-Miller et al., 2009). The present study agreed with this finding, indicating the significant difference in the actual blood glucose value between HM30 and control CSB at 30 min (HM30 = 6.93 mmol/L, control CSB = 7.76 mmol/L), 60 min (HM30 = 5.68 mmol/L, control CSB = 6.26 mmol/L), and 90 min (HM30 = 5.08 mmol/L, control CSB = 5.73 mmol/L). This phenomenon led to low-GI value for HM30 and medium-GI value for control CSB. The medium-GI value of white CSB was unexpected because in most studies, the GI value for white CSB was high and it was categorized as high-GI food (Su-Que et al., 2013). The variability in the GI value of white CSB could be explained by real differences among white CSB. The variability in the GI of control CSB in published studies could be attributed to differences in the physiology and genetics of subjects who participated in the GI trials (Su-Que et al., 2013). Given that the study protocol used by other studies for GI in white CSB was similar to that in the present work, the differences in GI values are therefore most likely due to subtle differences in the formulations of CSB and the properties of ingredients (Zhu, 2019).

### 3.6 Factors contributing to reduced glycemic index of HM30

The difference in GI values between HM30 and control CSB demonstrated the effect of HM on GI. Replacing wheat flour with HM reduced the content of rapidly digestible starch and increased the RS content (Zhang et al., 2006). HM30 had higher RS (22.08%) and slower starch digestion compared with control CSB. The presence of RS and dietary fiber in HM30 intervened with starch digestibility through their physicochemical interactions with HM composite starch, leading to the reduced GI of HM30 (Arp et al., 2018). The present study agreed with the results of Zhang et al. (2006), who reported that the substitution of HM into CSB reduced the GI. Substituting HM (30 g) into wheat flour (70 g) decreased the GI from 69.18 to 39.11, which delayed the glycemic response in healthy human subjects. Previous human studies on HM powder showed that it has blood glucose-lowering properties given that it delays the increase in postprandial blood glucose (Marlatt et al., 2018; Stewart et al., 2018).

HM30 was served an extra 55.03 g compared with control CSB to compensate for the higher amount of resistant starch and lower amount of available carbohydrates. Therefore, compared with control CSB, HM30 contains higher amount of protein and fat in preserving amount of 50 g available carbohydrate, thereby reducing the GI value of HM30 due to glycemic response-reducing effects of protein and fat (Brouns et al., 2005). Protein may interact with starch, forming a barrier surrounding the gelatinized starch (Jenkins et al., 1981). The barrier hinders the starch–enzyme interaction and reduces the starch digestibility. Fat can form inclusion complexes with amylose after steaming. These amylose complexes are largely enzyme resistant (Gelders et al., 2005), which may partially explain the reduced GI of HM30.

The lower GI of HM30 may also be attributed to its denser and more compact physical properties than control CSB (Collar et al., 2014). CSB produced using HM composite flour had higher values of firmness due to the colloidal properties of hydrocolloids (amylose and dietary fiber) in high-amylose maize starch (Wang et al., 2017). When HM was added, the development of gluten network was reduced and resulted in lower specific volume than control CSB (Li et al., 2020). According to Burton and Lightowler (2006), lower specific volume reduced the glycemic response and GI value of white bread; therefore, denser, and more compact CSB slowed down the rate of gastric emptying (Borczak et al., 2018). Moreover, the reduced gelatinization in the reduced volume of HM30 produced higher amount of resistant starch compared with that in control CSB (Haini et al., 2021). Substitution of digestible starch with RS reduced the glycemic response and the GI value (Zhang et al., 2006). The possible differences in the amount of RS does explain the lower GI value in HM30, which makes the GI value lower compared with those in previous studies.

## 4 Conclusion

Compositing HM into wheat flour produced lower GI in HM30 compared to control CSB. This could be due to the presence of high RS content, greater amount of protein and fat content in the CSB. Besides, the lower specific volume of HM could also contribute to reduced GI value and glycaemic response, suggesting that the denser and compact structure of HM30 slowed down the rate of gastric emptying in human subject. Overall, the findings of GI study in human indicated that application of HM as incorporated in the CSB product could be beneficial to be used as diet intervention in managing glycaemic control.

## Declarations

### Author contribution statement

Noraidah Haini: Performed the experiments; Analyzed and interpreted the data; Wrote the paper.

Lee Jau-Shya, Ramlah George Mohd Rosli, Hasmadi Mamat: Conceived and designed the experiments; Contributed reagents, materials, analysis tools or data.

### Funding statement

This work was supported by the 10.13039/501100002385Ministry of Higher Education Malaysia (MoHE) [FRGS/1/2017/STG/UMS/02/5].

### Data availability statement

The authors are unable or have chosen not to specify which data has been used.

### Declaration of interests statement

The authors declare no conflict of interest.

### Additional information

No additional information is available for this paper.
